# Complement C3 inhibition restores myasthenia gravis AChR antibody-mediated muscle pathophysiology

**DOI:** 10.1016/j.ebiom.2026.106322

**Published:** 2026-06-08

**Authors:** Yu-Fang Huang, Amol Keshavasa Bhandage, Miriam L. Fichtner, Anna Rostedt Punga

**Affiliations:** a Department of Medical Sciences, Clinical Neurophysiology, Uppsala University, Uppsala, Sweden; b Department of Neurology and Experimental Neurology, Charité - Universitätsmedizin Berlin, Berlin, Germany; c Koç University Research Center for Translational Medicine (KUTTAM), İstanbul, Türkiye

**Keywords:** Myasthenia gravis, Nicotinic acetylcholine receptors, VGCCs, Calcium signaling, C3, Complement inhibition

## Abstract

**Background:**

In the autoimmune disorder myasthenia gravis (MG), the nicotinic acetylcholine receptors (nAChRs) are the primary targets of pathogenic antibodies. While MG mechanisms have been extensively studied in animal models, functional insights into how antibody binding disrupts nAChR-dependent calcium signaling and the effects of complement inhibition in human muscle cells remain limited.

**Methods:**

We used real-time live-cell calcium imaging to assess the effects of cholinergic stimulation or inhibition on human muscle cells with pharmacological agents, sera from patients with acetylcholine receptor antibody seropositive (AChR+) MG, purified recombinant antibodies targeting α- and β-subunits, and a complement C3 inhibitor. Transcriptional changes in nAChR subunits, muscle markers, voltage-gated calcium channels (VGCCs), and complement-related genes were analysed by RT-qPCR. Immunocytochemistry and quantitative image analysis were performed to assess nAChR distribution and membrane attack complex (MAC) deposition.

**Findings:**

Cholinergic stimulation of human muscle cells activated nAChRs, resulting in membrane depolarisation, which in turn led to VGCC opening and calcium transients. MG-associated pathogenic antibodies, particularly those present in sera from patients with AChR + MG and pure recombinant AChR α-subunit-specific monoclonal antibody (mAb), but not β-subunit-specific, abolished choline-induced calcium responses in myotubes. α-subunit-specific mAb also induced transcriptional upregulation of nAChR subunits, muscle structural proteins, and complement components, and were associated with nAChR loss and MAC formation. Importantly, pharmacological inhibition of C3 activation restored calcium signaling, preserved nAChR distribution, and reduced MAC formation induced by α-subunit-specific mAb, implicating complement activation as a key driver of pathogenic effects.

**Interpretation:**

These findings indicate that targeting the α-subunit impairs nAChR-dependent calcium signaling and can induce complement activation. Disrupted signaling and reduced nAChR levels were effectively restored by C3 inhibition, which blocks multiple downstream pathways; thus, terminal complement activation leading to MAC formation is a suggested, but it may not be the only mechanism. Overall, the results highlight C3 as a promising upstream therapeutic target and support combining subunit-specific interventions with proximal complement blockade in MG.

**Funding:**

This work was supported by 10.13039/100007436Familjen Erling-Perssons Stiftelse (grant # 2022_0030 to ARP), the 10.13039/100003157Myasthenia Gravis Foundation of America, and the 10.13039/501100004359Swedish Research Council (grant # 2025-02779 to ARP). MLF part of the work was supported by a grant from the 10.13039/501100010275Deutsche Gesellschaft für Muskelkranke e.V. (DGM).

**Research in context**

**Evidence before this study**

Previous studies on the role of nicotinic acetylcholine receptors (nAChRs) in muscle cell excitation and on coupled calcium signaling in the regulation of muscle fatigability in myasthenia gravis (MG) have been limited and largely confined to animal models or simplified cell-based systems. Moreover, the physiological effects of subunit-specific pathogenic antibodies, whether naturally occurring in sera from patients with MG or in recombinant, purified forms, on the inhibition of muscle nAChR signaling remain poorly understood. Current therapies, including complement C5 inhibitors, improve clinical outcomes in MG but do not fully account for disease heterogeneity and have not been systematically evaluated for their mechanistic effects.

A more comprehensive understanding of subunit-specific antibody actions and the consequences of complement pathway inhibition is essential to inform precision treatment strategies for MG.

**Added value of this study**

Using a human muscle cell model, we demonstrate that cholinergic stimulation induces calcium transients via a cascade involving nAChR activation, membrane depolarisation, and subsequent opening of voltage-gated calcium channels, predominantly T-type Ca_V_3.2 channels. Furthermore, we show that a recombinant monoclonal antibody targeting the nAChR α-subunit, but not the β-subunit, completely abolishes cholinergic calcium transients, upregulates multiple complement components, and induces nAChR loss and membrane attack complex (MAC) formation, suggesting the dominant role of the α-subunit in receptor activation and implicating autoimmune damage via enhanced MAC formation. Sera from patients with AChR antibody-positive MG elicited similar effects to those observed with α-subunit–specific antibodies. Importantly, pharmacological inhibition of C3 effectively restored nAChR-mediated calcium responses. These findings identify the muscle nAChR α-subunit as a critical pathogenic target and highlight C3 as a promising upstream therapeutic node, supporting the rationale for subunit-specific interventions and proximal complement inhibition as precision medicine strategies in MG.

**Implications of all the available evidence**

These findings provide mechanistic insight into how MG-associated autoantibodies disrupt neuromuscular signaling and activate complement pathways in human muscle cells. By linking α-subunit inhibition to functional impairment and demonstrating that C3 blockade can restore these functions, this study supports the development of precision therapies that integrate antigen-specific approaches with proximal complement inhibition. Such strategies may enhance patient stratification and optimise treatment outcomes in MG, ultimately addressing the chronic, fatigable skeletal muscle weakness characteristic of the disease.

## Introduction

Myasthenia gravis (MG) is a chronic autoimmune disorder characterised by impaired neuromuscular transmission due to autoantibodies targeting components of the postsynaptic muscle membrane at the neuromuscular junction (NMJ).^1^ Antibodies against the nicotinic acetylcholine receptors (nAChRs) are most prevalent, detected in ∼85% of patients,^2^ while antibodies against others target proteins, such as muscle-specific kinase (MuSK) or lipoprotein receptor-related protein 4 (LRP4), are detected in approximately 8% and 3% of patients, respectively.^2^ The nAChRs are pentameric ligand-gated ion channels located on the postsynaptic membrane and mediate signal transmission from motor neurons to initiate muscle contraction. In adult skeletal muscles, nAChRs exhibit a subunit composition stoichiometry of (α1)_2_β1εδ, whereas fetal receptors contain γ instead of ε.^3^ The α-subunit represents the major pathogenic target in AChR seropositive (AChR+) MG, as its extracellular domain harbors the main immunogenic region (MIR), which contains overlapping epitopes targeted by more than 50% of autoantibodies in AChR + MG.^3^^,^^4^ The binding of these autoantibodies can lead to direct blocking of nAChRs, cross-linking-induced nAChR internalisation, and autoantibody-mediated complement activation resulting in damage of postsynaptic muscle membrane.^5^, ^6^, ^7^ Analysis of polyclonal antibodies in patient sera further demonstrates marked heterogeneity in pathogenic potential, with variable capacities to activate complement, induce receptor internalisation, or block acetylcholine binding across individuals and disease stages.^8^ The resulting loss of functional nAChRs on the muscle membrane diminishes acetylcholine (ACh)-induced endplate depolarisation and subsequent activation of voltage-gated calcium channels (VGCCs), impairing calcium influx and excitation-contraction coupling (ECC), a calcium-dependent process linking membrane excitation to muscle contraction.^9^ VGCCs, particularly L-type channels, play a central role in ECC by triggering calcium release from the sarcoplasmic reticulum,^10^ while T-type VGCCs contribute to calcium homeostasis and muscle cell integrity.^11^ Together, nAChR-mediated depolarisation and VGCC-driven calcium signaling form tightly coordinated steps essential for normal muscle physiology and function.^9^ Given the pivotal role of calcium ions as the essential mediators of secondary processes, intracellular calcium levels serve as a key determinant of muscle function and have been used as a reliable indicator to assess functionality of cell models.^12^^,^^13^

Complement activation to form membrane attack complex (MAC) is a key pathogenic mechanism in AChR + MG. Therapeutic inhibition of complement components, particularly C5, has demonstrated clinical efficacy by preventing C5 cleavage and subsequent MAC assembly.^14^ While inhibition of MAC formation is well established, the downstream cellular consequences of complement activation on nAChR-dependent calcium signaling in human skeletal muscle cells remain poorly understood. Prior studies have examined effects of MG sera on nAChR function in vivo using animal models and in vitro using human rhabdomyosarcoma-derived cell lines,^15^, ^16^, ^17^ but these models do not fully recapitulate human skeletal muscle physiology. Patient-derived monoclonal autoantibodies (mAbs) have provided important insights into the functional consequences of subunit-specific AChR recognition. Autoantibodies directed against the α-subunit can mediate all major pathogenic mechanisms, including complement activation, receptor blockade, and antigenic modulation.^18^ In contrast, antibodies specific for the β-subunit display a more restricted functional profile, predominantly driving complement activation and receptor modulation. Notably, several mAbs bind epitopes spanning the α–β subunit interface, indicating that some pathogenic antibodies may bridge neighboring subunits and thereby promote receptor clustering and enhanced effector activation.^18^ However, the functional implications of subunit-specific antibody binding for nAChR-dependent calcium signaling in human muscle cells remain unexplored.

This study aimed to evaluate the functional impact of MG-associated AChR antibodies in a human muscle cell model previously shown to form MAC upon exposure to sera from patients with AChR + MG.^19^ We assessed antibody-mediated effects on nAChR-dependent calcium signaling using real-time live-cell calcium imaging as well as transcriptional changes, nAChR distribution and MAC formation. Additionally, complement activation was pharmacologically inhibited to elucidate a role for MAC formation in neuromuscular transmission failure.

## Methods

### Human skeletal muscle myoblast culture and myotube differentiation

Primary human skeletal muscle myoblasts (HSMM; Cat. no. CC-2580, Lonza) were cultured as previously described.^19^ Briefly, HSMMs were expanded in growth medium (SkGMTM-2 Medium; Cat. no. CC-3246, Lonza) supplemented with SkGMTM-2 SingleQuotsTM Kit (Cat. no. CC-3244, Lonza) at a seeding density of 3500 cells/cm^2^ and sub-cultured for up to 10 passages. Myoblast fusion into myotubes was induced by incubation in differentiation medium consisting of DMEM/F-12 (Cat. no. 11330032, Gibco) supplemented with 2% horse serum (Cat. no. 16050122, Gibco), with medium changes every other day. Recombinant neural agrin (300 ng/mL; Cat. no. 550-AG-100, Bio-Techne) was added 3 days post-differentiation to mimics neuronal innervation feature on differentiated myotubes.

### Pharmacological agents

Carbamoylcholine chloride (Cat. no. 2810, Bio-Techne), tubocurarine hydrochloride pentahydrate (tubocurarine; Cat. no. T2379, Sigma–Aldrich), and potassium chloride (Cat. no. P5405, Sigma–Aldrich) were dissolved in phenol red-free Dulbecco’s Modified Eagle Medium/Nutrient Mixture F-12 (DMEM/F12; Cat. no. 21041025, Gibco). Stock solutions of benidipine (Cat. no. 3934, Bio-Techne) and nifedipine (Cat. no. N7634, Sigma–Aldrich) were prepared in dimethyl sulfoxide (DMSO) and diluted in phenol red-free DMEM/F12. Acetylcholine chloride (Cat. no. 2809, Bio-Techne), NNC 55-0396 dihydrochloride (Cat. no. 2268, Bio-Techne), and compstatin (Cat. no. 2585, Bio-Techne) were dissolved in water and diluted in phenol red-free DMEM/F12.

### Serum samples

MG sera were collected from three patients with AChR antibody-positive MG (confirmed by radioimmunoassay) with fatigable skeletal muscle weakness at Uppsala University Hospital (Supplementary Table S1). HC sera were obtained from three healthy donors at the Blood Center, Uppsala University Hospital. Blood samples were collected in additive-free tubes, allowed to coagulate at room temperature for ≥20 min, and centrifuged at 2200×*g* for 10 min at 4 °C. Serum aliquots were immediately stored at −80 °C until use.

### Recombinant purified monoclonal antibodies

AChR-specific, patient-derived mAbs (α-subunit-specific, mAb01b and β-subunit-specific, mAb09) were produced based on previously published variable region sequences kindly provided by Kevin C. O’Connor.^18^ Gene fragments encoding the variable heavy and light (κ for both) chain regions were synthesised (Eurofins) and cloned into human antibody expression vectors for the heavy and κ chain.^20^ Recombinant antibodies were expressed in HEK293A cells by transient transfection, purified from culture supernatants using protein G affinity chromatography,^21^ and dialysed against 1 × phosphate-buffered saline (PBS). Purified antibodies were aliquoted and stored at 4 °C until use.

### MG serum and monoclonal antibody treatments

For MG serum experiments, myotubes were incubated with human serum diluted 1:1 in DMEM/F-12 without horse serum supplementation for 24 h. For mAb experiments, myotubes were incubated with purified recombinant antibodies targeting AChR α- or β-subunits at a final concentration of 2 μg/mL for either 2 h or 24 h. These experiments were performed in differentiation medium consisting of DMEM/F-12 supplemented with 2% non-heat-inactivated horse serum as a complement source.

### RNA isolation and reverse transcription-quantitative PCR (RT-qPCR)

Total RNA was extracted using the RNeasy Mini Kit (Cat. no. 74104, Qiagen) and treated with DNase I (Cat. no. EN0521, Thermo Scientific). RNA was reverse-transcribed to cDNA using the iScript cDNA Synthesis Kit (Cat. no. 1708891, Bio-Rad). qPCR was performed on 4 ng cDNA using KAPA SYBR® FAST qPCR Master Mix (Cat. no. KK4622, Kapa Biosystems) and gene-specific primers (Supplementary Table S2)^22^^,^^23^ in QuantStudio 5 Real-Time PCR system (Thermofisher). The data are presented as relative mRNA expression (2ˆ(-ΔCT)) or normalised expression (2ˆ(-ΔΔCT)), with the geometric mean of four reference genes (GAPDH, TBP, IPO8, and RPLP0).

### Protein lysate preparation

Cells were washed with cold Dulbecco’s Phosphate-Buffered Saline (DPBS; Cat. no. 14190-144, Gibco) and lysed in RIPA buffer (Cat. no. 89901, Thermo Fisher Scientific) containing Halt Protease and Phosphatase Inhibitor Cocktail (Cat. No. 78442, Thermo Fisher Scientific). Lysates were gently rotated for 30 min at 4 °C, centrifuged at 14,000×*g* for 10 min at 4 °C, and supernatants were stored at −80 °C. Protein concentration was measured using the DC Protein Assay kit (Cat. no. 5000112, Bio-Rad) at 750 nm on CLARIOstar Plus microplate reader (BMG Labtech).

### Capillary-based western assay

Capillary western analysis was performed using Jess Automated Western Blot System (ProteinSimple) with a 12-230 kDa Fluorescence Separation Module (ProteinSimple SM-W003). Lysates and recombinant human nicotinic acetylcholine receptor alpha 1 (CHRNA1) protein (positive control; Cat. no. ab235737, Abcam) were diluted to 60 μg/mL in 0.1× Sample Buffer with 5 × Fluorescent Master Mix containing DTT, heated at 95 °C for 5 min, and then cooled on ice. After denaturation, samples, along with the blocking reagent, CHRNA1 primary antibody (Cat. no. NBP2-81070, Novus Biologicals), Rabbit anti-Rat IgG (H + L) secondary antibody, biotin (Cat. no. 31834, Invitrogen), Streptavidin-HRP, and chemiluminescent substrate, were loaded into designated wells. A biotinylated molecular weight ladder was included. The plate was centrifuged at 1000×*g* for 5 min at room temperature and processed for automated separation, electrophoresis, and immunodetection in the capillary system.

### Live cell Ca^2+^ imaging

HSMMs were cultured in μ-Slide I Luer chambers (Cat. no. 80196, Micromedic) in growth medium and differentiated for 7 days. Time-lapse calcium imaging was performed as previously described.^24^ Cells were incubated with 4 μM Fluo-8H calcium indicator (Cat. no. AATB21091, AAT Bioquest) for 60 min at 37 °C, then washed with DMEM/F12 before imaging. Real-time fluorescence (confocal) images were acquired every 2.5-s interval using a Stellaris 5 confocal microscope (Leica) with a 10× objective. Baseline recording was conducted during the initial 3 min of each experiment. Drugs were applied using a peristaltic pump system (Masterflex) at specific periods and concentrations indicated in respective traces in the figures. The fluorescence intensity was extracted and quantified by manually marking single myotubes from the series of time-lapsed images using ImageJ software and pseudo-colored images were created to represent fluorescence intensity scale. Data were presented as normalised fluorescence intensity (F/F_B_, with B as baseline) or maximal intensity (F/F_B_)_max_.

### Membrane potential measurement

HSMMs were differentiated into the chambers as described above. The potential-sensitive dye (bis-(1,3-dibutylbarbituric acid) trimethine oxonol, DiBAC_4_(3); Cat. no. B438, Invitrogen) was prepared according to.^25^ DiBAC_4_(3) is a voltage-sensitive fluorescent probe that enters and accumulates in the depolarised cells, with a sensitivity of ∼1% fluorescence change per 1 mV shift.^25^^,^^26^ DiBAC_4_(3) and its responses are characterised in electrically excitable cells showing a linear relationship between the changes in fluorescence intensity and cell membrane potential.^27^ Myotubes were washed with PBS containing 1 mM of calcium and magnesium, and then incubated with 5 μM DiBAC_4_(3) for 30 min at 37 °C. Image acquisition and fluorescence intensity quantification were performed as described above.

### Immunocytochemistry

HSMMs were cultured in μ-Slide 8 well high chamber slides (Cat. no. 80809, Micromedic) in growth medium and differentiated for 7 days. Cells were rinsed with warm PBS and incubated with tetramethylrhodamine-conjugated α-bungarotoxin (Cat. no. T0195, Sigma–Aldrich) for 45 min at 37 °C to label nAChRs. Cells were fixed in 4% paraformaldehyde (Cat. no. J19943.K2, Thermo Fisher Scientific) for 30 min at room temperature, washed with PBS, and permeabilised with 0.25% Triton X-100 (Cat. no. X100, Sigma–Aldrich) in PBS for 10 min. Non-specific binding was blocked with 1% bovine serum albumin (BSA; Cat. no. A7906, Sigma–Aldrich) in PBS for 1 h at room temperature. Cells were incubated with primary antibodies against desmin (Cat. no. ab32362, Abcam), sarcomeric α-actinin (Cat. no. ab9465, Abcam) or complement component C5b-9 (MAC; Cat. no. DIA 011-01-02, BioPorto) overnight at 4 °C. After washing, appropriate Alexa Fluor 405- or 488-conjugated secondary antibodies (Cat. no. ab175649 and ab150113, Abcam) were applied in PBS containing 1% BSA for 1 h at room temperature in the dark. Nuclei were stained with DAPI. Images were acquired using a confocal microscope (Zeiss LSM700) with 20× or 63× objectives.

### Image analysis and quantification of nAChR and MAC

Fluorescence images were analysed using ImageJ software. For each image, channels corresponding to nAChR, C5b-9 (MAC), and desmin were separated and thresholded using the Otsu method to distinguish signal from background. Thresholded images were converted into binary masks, and signal areas were measured. Overlapping areas between nAChR and desmin, as well as between C5b-9 (MAC) and desmin, were quantified using the Image Calculator function. Desmin staining was used to define the myotube area, ensuring that only signals associated with myotubes were analysed while excluding off-muscle artifacts. Results were expressed as nAChR-Desmin overlap area/Desmin area (%) and C5b-9 (MAC)-Desmin overlap area/Desmin area (%). For each group, 18 images from three independent experiments were analysed (6 images per experiment).

### Cell viability assay

Cell viability was assessed using the trypan blue exclusion method. HSMMs were cultured and differentiated in 24-well plates for 7 days. After mAbs treatment, cells were harvested and mixed with trypan blue (Cat. no. 15250061, Thermo Fisher Scientific), then loaded into counting slides. Viable and non-viable cells were quantified using a Countess 3 Automated Cell Counter (Thermo Fisher Scientific). Viability was calculated as the percentage of viable cells to the total number of cells counted.

### Reagent validation

Cell validation was confirmed by the supplier Lonza with the batch-specific certificate of analysis (CoA; batch no. 21TL138913). According to the CoA, the cells were mycoplasma-free and sterile, exhibited high viability (97%), and myogenic identity based on desmin-positive differentiation. Cell cultures were regularly tested for mycoplasma by qPCR using the following primers: forward primer (TGCACCATCTGTCACTCTGTTAACCTC) and reverse primer (GGGAGCAAACAGGATTAGATACCCT); no amplification was detected. Commercial antibodies (Supplementary Table S3) were validated by the manufacturers and used according to the recommended conditions. The monoclonal antibodies mAb01b and mAb09 were produced for this study based on previous publication.^18^

### Ethics

The study was approved by the Swedish Ethical Review Authority (permit numbers 2020-03049, 2023-01455-02, and 2024-02500-01). Written informed consent was obtained from all patients with MG and healthy controls (HCs).

### Data mining and statistics

Sample size (n) represents independent biological experiments and/or individual donor sera. Statistical analyses were performed in GraphPad Prism software v10 (GraphPad Software, USA). Normality of all datasets was assessed using the Shapiro–Wilk test. For unpaired comparisons, normally distributed data were analysed using an unpaired t-test, whereas non-normally distributed data were analysed using the Mann–Whitney test. For paired datasets, a paired t-test was used for normally distributed data, and the Wilcoxon signed-rank test was used when normality was not met. For comparisons involving more than two groups, one-way ANOVA was used for normally distributed data, while non-normally distributed data were analysed using the Kruskal–Wallis test followed by Dunn’s post hoc correction. A p-value < 0.05 was considered statistically significant.

### Role of funders

The funders had no role in study design, data collection, analysis, interpretation, or manuscript preparation.

## Results

### Characterisation of differentiated human myotubes and nAChR expression

**Fig. 1:**
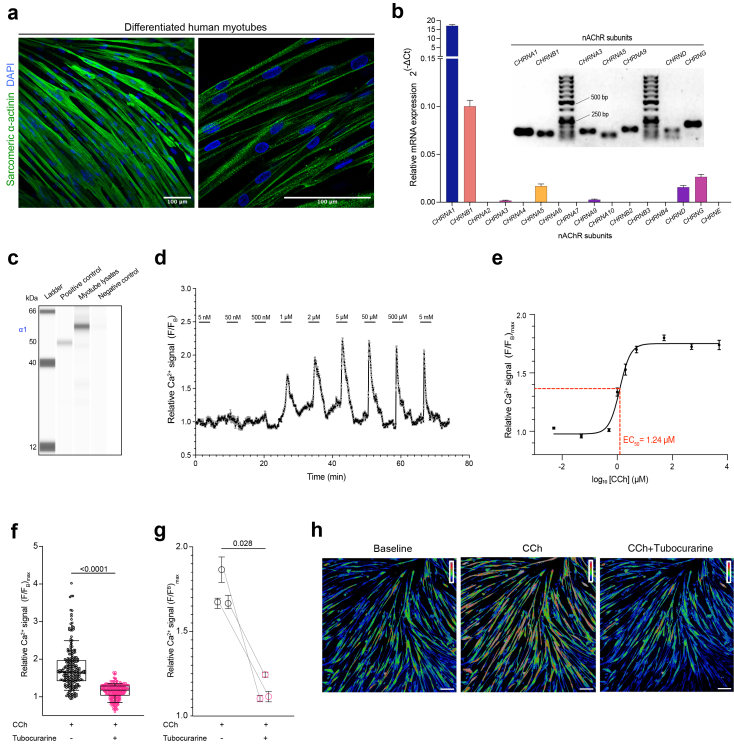
**Choline-induced calcium responses are exhibited through activation of nicotinic acetylcholine receptors (nAChRs) in human myotubes. (a)** Differentiated human myotubes stained for sarcomeric α-actinin (green) and nuclei (DAPI, blue), with 20× (left) and 63× (right) magnifications. Scale bar, 100 μm **(b)** Relative mRNA expression of nAChR subunits. Inset presents bands of DNA amplicons at correct sizes for the detected subunits along with DNA ladder. mRNA expression data are presented as 2^(−ΔCt)^ with mean ± SEM. **(c)** Detection of human AChR α1 protein in positive control (recombinant pure protein) and myotube lysates by capillary-based western assay. **(d)** A representative trace of calcium responses in human myotubes upon sequential application of carbamoylcholine chloride (CCh) with increasing concentrations and application periods indicated by horizontal lines. Data presented are mean ± SEM of relative intensity $\left(F/F_{B}\right)$ obtained from at least 30 individual myotubes. **(e)** EC_50_ curve for CCh concentrations ranging from 5 nM to 5 mM. Each data point represents mean ± SEM of maximal intensity ((F/F_B_)_max_) at a particular concentration (n = 6 experiments, a total of 1800 myotubes). The concentration-response curve was fitted using nonlinear regression and EC_50_ value (indicated by red lines) for CCh was determined by Hill’s Slope equation. Quantification of CCh-induced calcium responses in the absence or presence of nAChR antagonist tubocurarine, shown as **(f)** individual myotubes (n = 3 experiments, a total of 220 myotubes per group; box-and-whisker plots with median, interquartile range, and 10th–90th percentiles; Wilcoxon matched-pairs signed-rank test) and **(g)** average values (n = 3 experiments; mean ± SEM; paired data connected by lines; paired t-test). **(h)** Representative pseudocolored micrograph of human myotubes loaded with calcium indicator Fluo-8H under three conditions: baseline (no stimulation; left), stimulation with 50 μM CCh (middle), and co-application of CCh and 100 μM tubocurarine (CCh + Tubocurarine; right). The pseudocolor scale bar at top right corner indicates fluorescence intensity levels, with blue as the lowest and red as the highest. Scale bar, 100 μm. Exact p-values are indicated in the figure (ns = not significant).

Differentiation of human skeletal muscle myoblasts into myotubes was confirmed by immunocytochemistry for sarcomeric α-actinin, a Z-disc structural protein serves as a marker of sarcomere formation in differentiated skeletal muscle cells.^28^ The elongated, multinucleated myotubes exhibited sarcomeric α-actinin, indicating the presence of contractile apparatus components characteristic of differentiated skeletal muscle (Fig. 1a). To characterise the nAChR subunit composition in myotubes, mRNA expression analysis of all 16 nAChR subunits was performed. The *CHRNA1* (α1) exhibited the highest expression, followed by *CHRNB1* (β1) (Fig. 1b), consistent with the characteristic profile of muscle-type nAChRs. Transcripts of *CHRNA5* (α5), *CHRND* (δ), and *CHRNG* (γ) was also detected, while *CHRNA3* (α3) and *CHRNA9* (α9) were present at low levels, and other subunits were undetectable (Fig. 1b). The mRNA expression was confirmed by detection of correct size DNA amplicons on agarose gel (Fig. 1b). Protein expression of nAChR α1 was confirmed using capillary Western immunoassay (Fig. 1c).

### Choline-induced calcium responses are exhibited through nAChR activation

To assess the functionality of nAChRs in human muscle cells, the effect of cholinergic agonist was measured on intracellular calcium responses using real-time calcium imaging. ACh is rapidly hydrolysed due to its low enzymatic stability in the cell culture and in vivo. Carbamoylcholine chloride (CCh), a cholinergic agonist structurally similar to ACh that also activates muscle-type nAChRs^29^ but is not prone to enzymatic degradation and exhibits longer duration of action.^30^ Both ACh and CCh responded with comparable calcium transients in myotubes (Supplementary Fig. S1), validating CCh as a physiologically relevant and practically stable substitute for long duration experiments. Sequential application of CCh (5 nM–5 mM) elicited robust calcium transients starting at 1 μM, with a sigmoidal, dose-response curve (Fig. 1d and e, Supplementary video). An EC_50_ value of 1.24 μM was determined using the Hill slope equation. Based on these findings, concentrations of 50–500 μM CCh were selected to ensure maximal activation in subsequent experiments.

To verify that calcium responses were mediated through the activation of nAChRs, tubocurarine, a specific and competitive nAChR antagonist, was co-applied with CCh after a control recording. Tubocurarine significantly reduced maximal calcium responses induced by CCh (Fig. 1f, n = 3; CCh: median = 1.7, 10th–90th percentiles = 1.2–2.5; CCh + tubocurarine: median = 1.2, 10th–90th percentiles = 0.8–1.3; Wilcoxon matched-pairs signed-rank test, p-value < 0.0001; Fig. 1g, n = 3; CCh: mean ± SEM = 1.7 ± 0.07; CCh + tubocurarine: mean ± SEM = 1.2 ± 0.05; paired t-test, p-value = 0.028), excluding involvement of metabotropic AChRs. Pseudocolor images illustrated fluorescence intensity changes: baseline myotubes displayed low-to-moderate intensities (blue-green), whereas CCh stimulation induced strong signals (orange-red), which were attenuated by tubocurarine (Fig. 1h). Overall, these results confirm that choline-induced calcium responses in human myotubes are mediated through functional nAChR activation.

### nAChR activation depolarised membrane potential

**Fig. 2:**
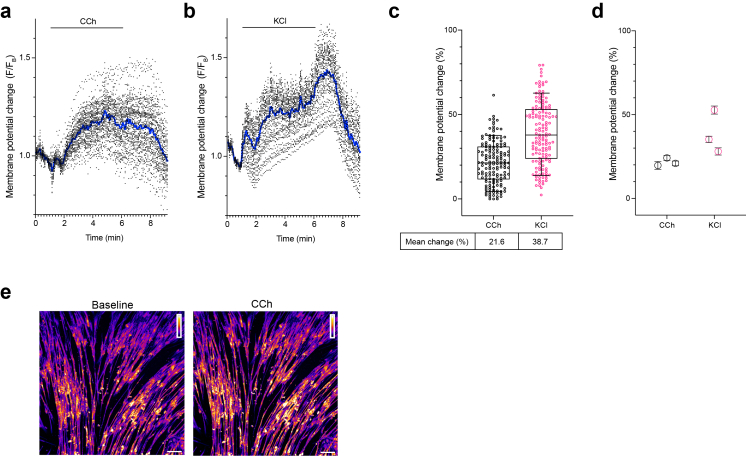
**Visualisation of DiBAC_4_(3)intensity changes as indicator of membrane depolarisation in human myotubes upon cholinergic stimulation.** Membrane potential changes were assessed by voltage-sensitive dye DiBAC_4_(3). Traces of DiBAC_4_(3) intensity from 50 individual myotubes with **(a)** carbamoylcholine chloride (50 μM CCh) or with **(b)** potassium chloride (150 mM KCl), application period is indicated by horizontal lines. Data are presented as relative intensity $\left(F/F_{B}\right)$ with blue line representing mean. Membrane potential change (%) from myotubes responded to CCh and KCl, determined as 1% change in intensity $\left(F/F_{B}\right)$ as 1 mV change. Data are presented as **(c)** individual myotubes (n = 3 experiments, a total of 150 per group; box-and-whisker plots with median, interquartile range, and 10th–90th percentiles) and **(d)** average values (n = 3 experiments; mean ± SEM). **(e)** Representative pseudocolor images of DiBAC_4_(3) fluorescence intensity of myotubes before (Baseline, left) and after CCh application (CCh, right). The pseudocolor scale bar at top right corner indicates intensities, the highest in bright yellow (regions of greater membrane depolarisation) and the lowest in dark purple. Scale bar, 100 μm.

Since nAChR activation permits influx of cations (primarily Na^+^), we investigated whether CCh stimulation depolarises myotube membrane. Membrane potential dynamics were assessed using DiBAC_4_(3). Normalised fluorescence (F/F_B_) traces revealed that both CCh and KCl (a positive control) induced depolarisation, reaching clear maxima followed by gradual decline (Fig. 2a and b). Quantification showed mean fluorescence increases of 21.6% for CCh and 38.7% for KCl (Fig. 2c and d), corresponding to approximately 21.6 mV and 38.7 mV depolarisation of the membrane potential respectively. Pseudocolor images of DiBAC_4_(3) fluorescence intensity, with CCh-treated myotubes exhibiting increased fluorescence intensity compared to baseline, consistent with membrane depolarisation (Fig. 2e).

### Choline-induced depolarisation activated VGCCs to drive calcium responses

**Fig. 3:**
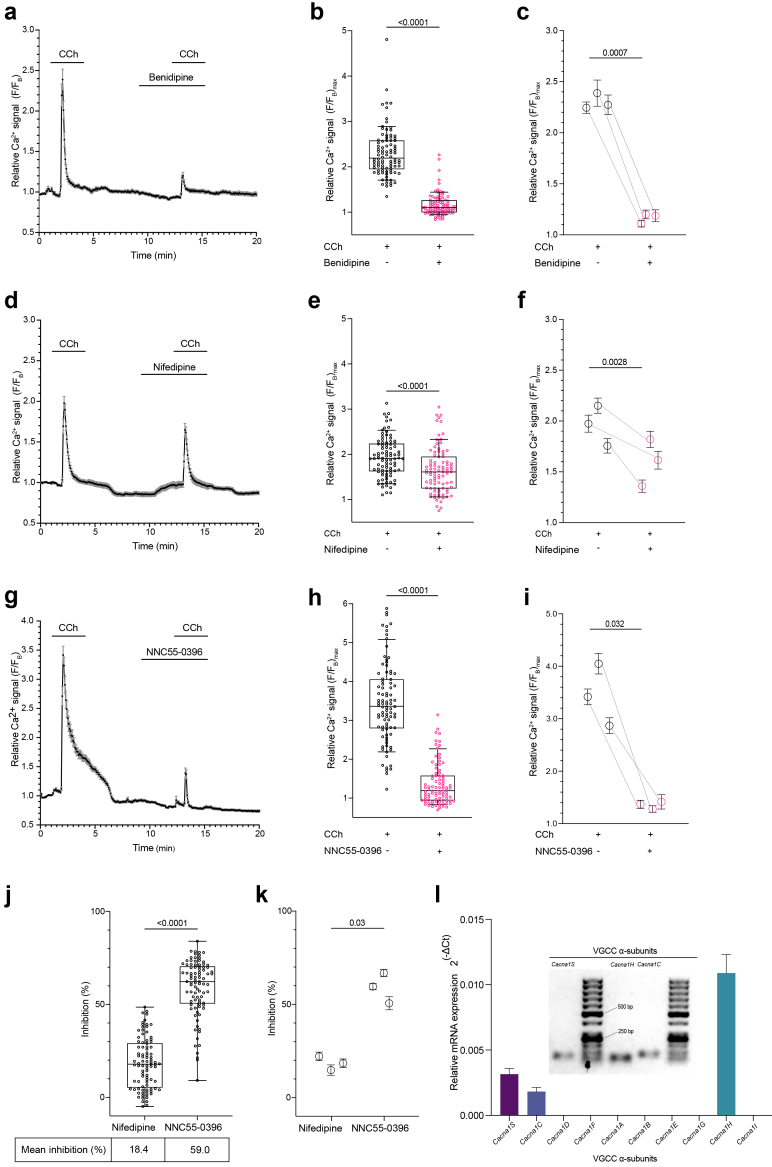
**Choline-induced calcium responses in human muscle cells are driven by voltage-gated calcium channels (VGCCs).** CCh (500 μM) induced calcium response in human myotubes, followed by co-application with VGCC inhibitors, **(a–c)** benidipine (10 μM) or **(d–f)** nifedipine (10 μM) or **(g–i)** NNC 55-0396 dihydrochloride (NNC 55-0396, 10 μM). The representative traces of fluo-8H loaded cells presented as mean ± SEM of relative intensity $\left(F/F_{B}\right)$ from 30 individual myotubes. The applications periods are indicated by horizontal lines. Quantification is shown as **(b, e, h)** individual myotubes (n = 3 experiments, a total of 90 per group; box-and-whisker plots with median, interquartile range, and 10th–90th percentiles; Wilcoxon matched-pairs signed rank test) and **(c, f, i)** average values (n = 3 experiments; mean ± SEM; paired data connected by lines; paired t-test). Percentage inhibition of CCh-induced calcium responses following nifedipine (L-type) or NNC 55-0396 (T-type) blockade was calculated by setting CCh responses to 100% and presenting inhibitor responses relative to CCh. Data are presented as **(j)** individual myotubes (box-and-whisker plots with median, interquartile range, and 10th–90th percentiles; Mann–Whitney test) and **(k)** average values (n = 3 experiments; mean ± SEM; unpaired t-test). **(l)** Relative mRNA expression of VGCC a-subunits in human myotubes. Data are presented as mean ± SEM. The inset shows bands of DNA amplicons for VGCC α-subunits at the correct sizes, with a DNA ladder indicating 500 and 200 base pairs (bp). Exact p-values are indicated in the figure (ns = not significant).

Since CCh induced membrane depolarisation, it was further investigated whether VGCCs were activated. Pharmacological inhibition with benidipine, an antagonist that blocks all subtypes of VGCCs, completely abolished CCh-induced calcium responses (Fig. 3a–c; Fig. 3b, n = 3; CCh: median = 2.2, 10th–90th percentiles = 1.7–2.9; CCh + benidipine: median = 1.1, 10th–90th percentiles = 0.9–1.4; Wilcoxon matched-pairs signed-rank test, p-value < 0.0001; Fig. 3c, n = 3; CCh: mean ± SEM = 2.3 ± 0.04; CCh + benidipine: mean ± SEM = 1.2 ± 0.03; paired t-test, p-value = 0.0007). To examine the contribution of specific channel subtypes, selective inhibitors were applied. Inhibition of L-type (high-voltage-activated) channels with nifedipine partially but significantly reduced the calcium response (Fig. 3d–f; Fig. 3e, n = 3; CCh: median = 1.9, 10th–90th percentiles = 1.4–2.6; CCh + nifedipine: median = 1.5, 10th–90th percentiles = 1.1–2.2; Wilcoxon matched-pairs signed-rank test, p-value < 0.0001; Fig. 3f, n = 3; CCh: mean ± SEM = 2.0 ± 0.1 CCh + nifedipine: mean ± SEM = 1.6 ± 0.1; paired t-test, p-value = 0.0028), whereas inhibition of T-type (low-voltage-activated) channels with NNC 55-0396 resulted in a markedly stronger suppression of the calcium response (Fig. 3g–i; Fig. 3h, n = 3; CCh: median = 3.4, 10th–90th percentiles = 2.2–5.1; CCh + NNC 55-0396: median = 1.2, 10th–90th percentiles = 0.8–2.3; Wilcoxon matched-pairs signed-rank test, p-value < 0.0001; Fig. 3i, n = 3; CCh: mean ± SEM = 3.4 ± 0.3 CCh + NNC 55-0396: mean ± SEM = 1.4 ± 0.04; paired t-test, p-value = 0.032). To assess subtype-specific inhibition, the percentage inhibition was calculated, revealing 18.4% inhibition with nifedipine and 59.0% inhibition with NNC 55-0396 (Fig. 3j and k; Fig. 3j, n = 3; nifedipine: median = 17.9, 10th–90th percentiles = 1.3–39.0; NNC 55-0396: median = 62.3, 10th–90th percentiles = 36.3–75.1; Mann–Whitney test, p-value < 0.0001; Fig. 3k, n = 3; nifedipine: mean ± SEM = 18.8 ± 2.1; NNC 55-0396: mean ± SEM = 60.0 ± 5.1; unpaired t-test, p-value = 0.03). Since subunits determine the functionality and pharmacological properties of VGCCs, we investigated VGCC subunit expression profile in human myotubes. This analysis revealed expression of *Cacna1S* (Ca_V_1.1), and *Cacna1C* (Ca_V_1.2), which form L-type channels, and *Cacna1H* (Ca_V_3.2), which form T-type channels (Fig. 3l), confirmed by correct-size amplicons on agarose gel (Fig. 3l). The *Cacna1H* (Ca_V_3.2) had the highest expression levels in human myotubes, whereas other subunits were undetectable. These results suggest that CCh-induced calcium influx is mediated by VGCC activation following nAChR-driven depolarisation, with a prominent role for T-type channels.

### MG-associated antibodies suppress nAChR-mediated calcium signaling

**Fig. 4:**
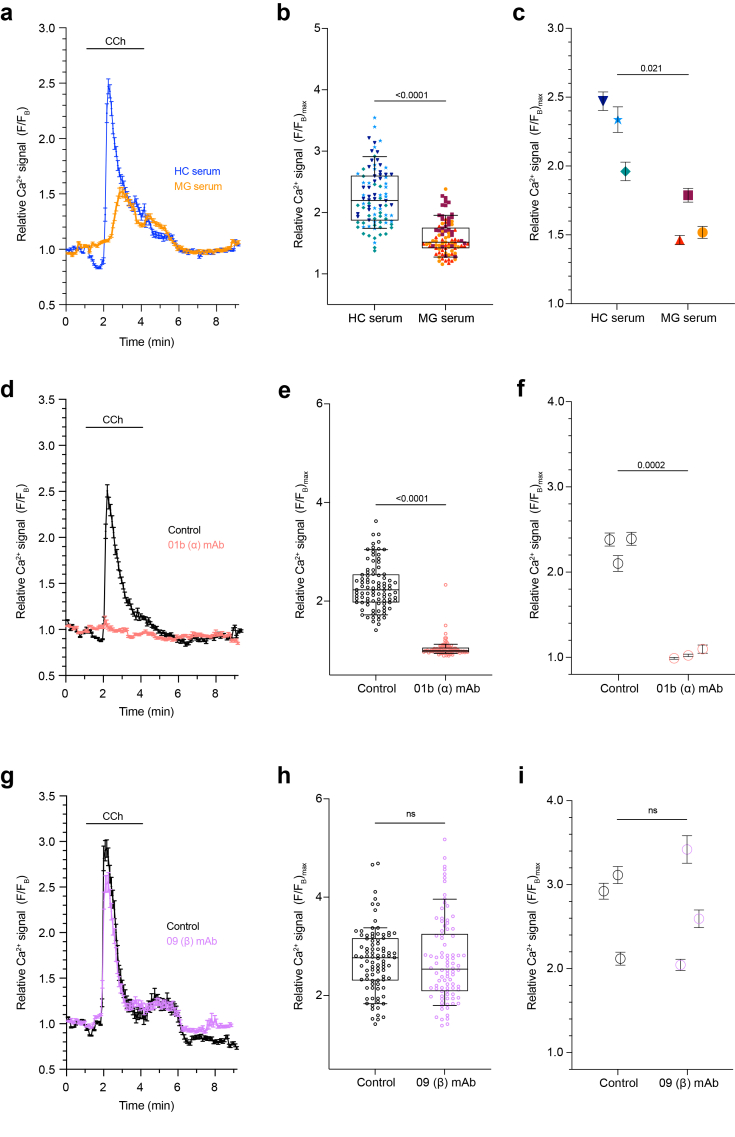
**Myasthenia gravis (MG)-associated antibodies inhibit CCh-induced calcium responses. (a)** Representative traces of CCh-induced calcium responses in healthy control (HC) serum-treated (blue) and myasthenia gravis (MG) serum-treated (orange) myotube groups. Application of CCh is indicated by a horizontal line. Data are presented as mean ± SEM of relative intensity $\left(F/F_{B}\right)$ from 30 individual myotubes in each group. Quantification of maximal CCh-induced calcium responses ((F/F_B_)_max_) is shown as **(b)** individual myotubes, with each symbol representing data from treatment with a particular HC or MG serum (n = 3 donors, a total of 90 per group; box-and-whisker plots with median, interquartile range, and 10th–90th percentiles; Mann–Whitney test) and **(c)** average values (n = 3 experiments; mean ± SEM; unpaired t-test). Representative traces and quantifications of myotubes treated with recombinant monoclonal antibodies (mAbs; 2 μg/mL) directed against nAChR subunit **(d–f)** α (01b mAb) or **(g–i)** β (09 mAb) on CCh-induced calcium transients. Traces are presented as mean $\left(F/F_{B}\right)$ ± SEM from 30 individual myotubes in each group. Quantification as maximal CCh-induced calcium responses ((F/F_B_)_max_) is shown as (e, h) individual myotubes (n = 3 experiments; a total of 90 per group; box-and-whisker plots with median, interquartile range, and 10th–90th percentiles; Mann–Whitney test) and (f, i) average values (n = 3 experiments; mean ± SEM; unpaired t-test). Exact p-values are indicated in the figure (ns = not significant).

To assess the pathogenic impact of MG-associated AChR antibodies, myotubes were incubated for 24 h with sera from patients with generalised AChR + MG (MGFA class II-III) or age- and sex-matched HCs (Supplementary Table S1). Myotubes exposed to HC serum exhibited robust calcium transients upon CCh stimulation, whereas sera from all three patients with AChR + MG markedly suppressed these responses (Fig. 4a–c; Fig. 4b, n = 3; HC: median = 2.2, 10th–90th percentiles = 1.7–2.9; MG: median = 1.5, 10th–90th percentiles = 1.3–1.9; Mann–Whitney test, p-value < 0.0001; Fig. 4c, n = 3; HC: mean ± SEM = 2.3 ± 0.2; MG: mean ± SEM = 1.6 ± 0.1; unpaired t-test, p-value = 0.021; Supplementary Fig. S2). Given that nAChR autoantibodies derived from patients with MG are polyclonal, we next dissected subunit-specific effects using mAbs targeting the nAChR α- and β-subunits (AChR01b and AChR09).^18^ After 24 h incubation, mAb against the α-subunit completely abolished CCh-induced calcium responses (Fig. 4d–f; Fig. 4e, n = 3; control: median = 2.2, 10th–90th percentiles = 1.7–3.0; 01b (α) mAb: median = 1.0, 10th–90th percentiles = 0.9–1.1; Mann–Whitney test, p-value < 0.0001; Fig. 4f, n = 3; control: mean ± SEM = 2.3 ± 0.1; 01b (α) mAb: mean ± SEM = 1.0 ± 0.03; unpaired t-test, p-value = 0.0002), whereas β-subunit mAb had no significant effect (Fig. 4g–i). These findings demonstrate that MG-associated antibodies impair nAChR-dependent calcium responses in human myotubes and specifically highlight the critical role of α-subunit targeting in this pathogenic mechanism.

### Transcriptional alterations upon exposure to MG-associated antibodies

**Fig. 5:**
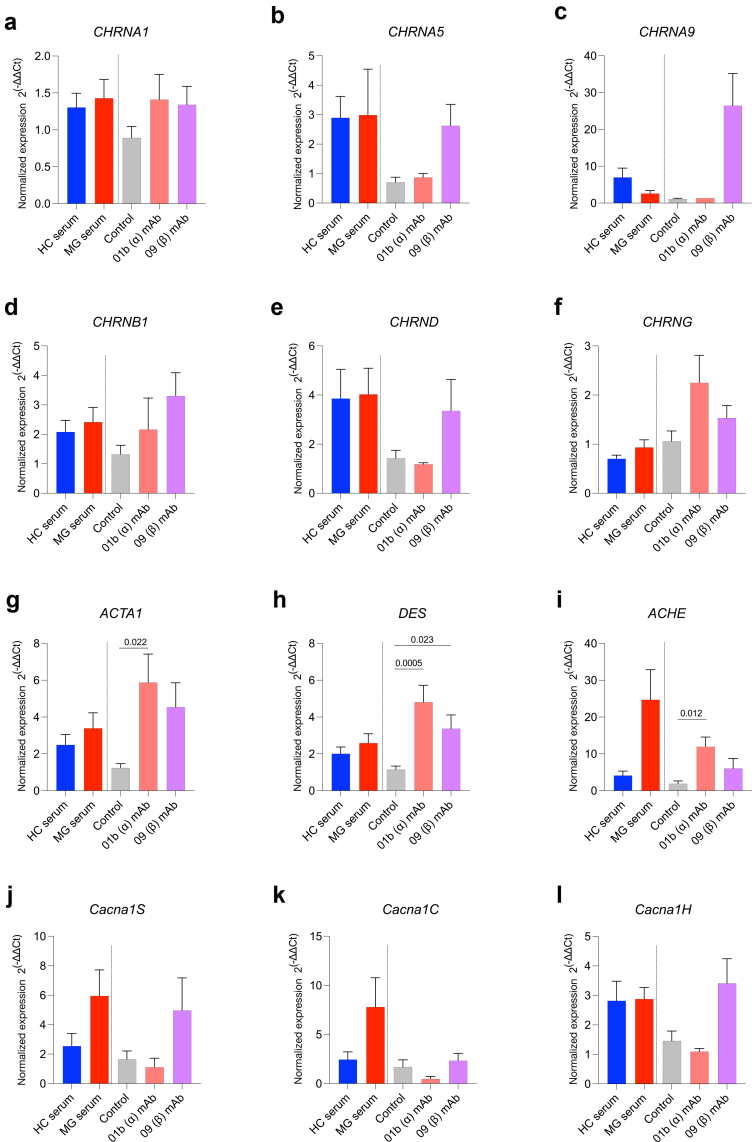
**Transcriptional changes in the expression of nAChR and VGCC subunits, muscle cell cytoskeletal proteins.** Relative mRNA expression of **(a–f)** nAChR subunits, **(g–h)** muscle cytoskeletal structural markers, **(i)** acetylcholinesterase (AChE), and **(j**–**l)** VGCC subunits in human myotubes treated with healthy control (HC) serum, MG serum, or recombinant monoclonal AChR antibodies targeting the α (01b) or β (09) subunits, shown as bar charts. Normalisation was performed to the control group. Data are presented as 2^(−ΔΔCt)^ with mean ± SEM. Statistical comparisons were performed separately for HC serum versus MG serum group (n = 12 experiments; Mann–Whitney test or unpaired t-test, based on the normality of data), and control versus α (01 b) or β (09) mAb group (n = 12 experiments; Kruskal–Wallis test followed by Dunn’s multiple comparisons test or one-way ANOVA, based on the normality of data). All comparisons were performed, but only those with statistical significance are annotated. Exact p-values are indicated in the figure (ns = not significant).

To determine whether functional blockade by pathogenic antibodies is accompanied by transcriptional changes, mRNA expression of nAChR subunits, VGCC subunits, and skeletal muscle markers was analysed in myotubes exposed to MG sera or recombinant mAbs. Because serum composition differed between serum- and mAb-treated groups, comparisons were performed separately. Transcripts of *CHRNA1* (α1), *CHRNB1* (β1), and *CHRNG* (γ) showed a tendency of upregulation in both mAb-treated myotubes (Fig. 5a, d, 5f), whereas *CHRNA5* (α5), *CHRNA9* (α9), and *CHRND* (δ) had a tendency of upregulation in β-subunit-specific mAb-treated myotubes (Fig. 5b, c, e). *ACTA1* (α-actin) and *ACHE* (acetylcholinesterase; AChE) were increased in α-subunit-specific mAb-treated myotubes (Fig. 5g and i, *ACTA1,* n = 12, control: mean ± SEM = 1.2 ± 0.2, 01b (α) mAb: mean ± SEM = 5.8 ± 1.5, 09 (β) mAb: mean ± SEM = 4.5 ± 1.3; *ACHE,* n = 12, control: mean ± SEM = 1.9 ± 0.7, 01b (α) mAb: mean ± SEM = 12.0 ± 2.6, 09 (β) mAb: mean ± SEM = 6.0 ± 2.7; Kruskal–Wallis test, p-value = 0.022 and 0.012, respectively), whereas *DES* (desmin) was elevated in both α- and β-subunit mAb-treated myotubes (Fig. 5h, n = 12, control: mean ± SEM = 1.2 ± 0.2, 01b (α) mAb: mean ± SEM = 4.8 ± 0.9, 09 (β) mAb: mean ± SEM = 3.4 ± 0.7; Kruskal–Wallis test, p-value = 0.0005 and 0.023, respectively). VGCC subunits revealed non-significant trends of increase in transcripts of *Cacna1S* (Ca_V_1.1) and *Cacna1C* (Ca_V_1.2) with MG sera exposure, whereas *Cacna1H* (Ca_V_3.2) had a trend of increase with β-subunit-specific mAb exposure (Fig. 5j–l). In summary, recombinant mAbs induced more pronounced transcriptional changes than polyclonal antibodies in MG serum.

### α-Subunit-Specific mAbs upregulate complement components

**Fig. 6:**
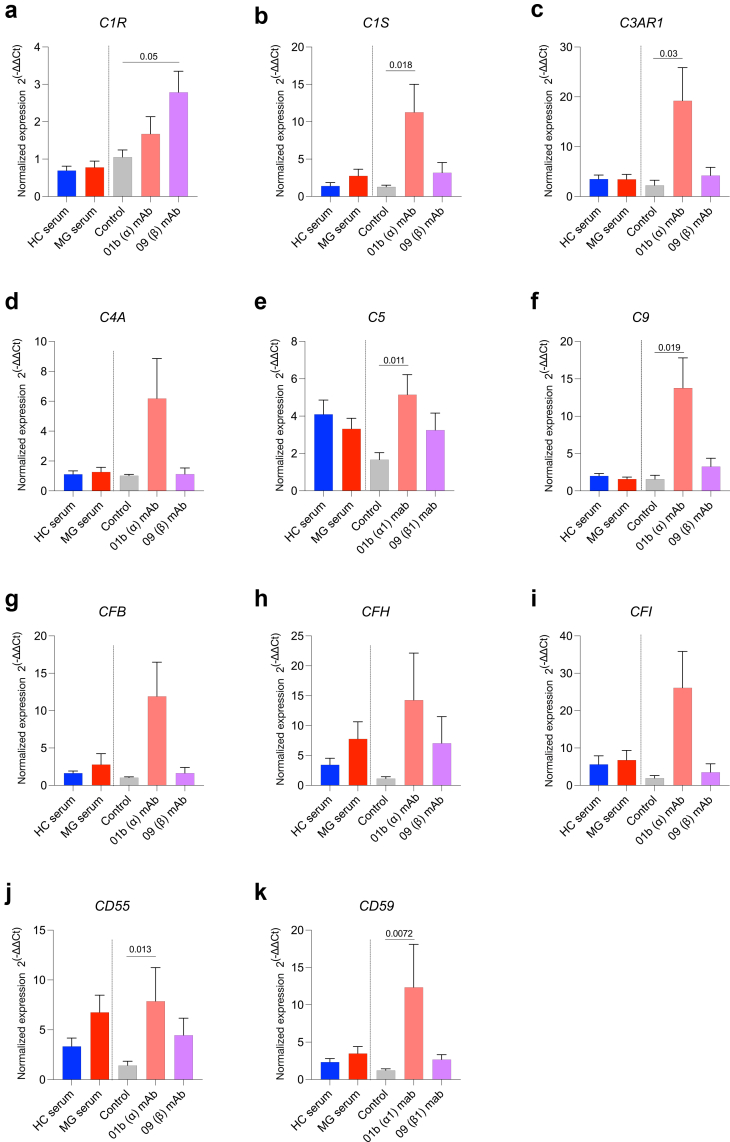
**nAChR α-subunit-specific mAbs upregulate expression of complement components.** Relative mRNA expression of **(a–i)** complement components and **(j**–**k)** complement regulatory proteins in human myotubes treated with healthy control (HC) serum, myasthenia gravis (MG) serum, or recombinant monoclonal AChR antibodies targeting the α (01b) or β (09) subunits, shown as bar charts. Normalisation was performed to the control group. Data are presented as 2^(−ΔΔCt)^ with mean ± SEM. Statistical comparisons were performed separately for HC serum versus MG serum group (n = 12; Mann–Whitney test or unpaired t-test, based on the normality of data), and control versus α (01b) or β (09) mAb group (n = 12 experiments; Kruskal–Wallis test followed by Dunn’s multiple comparisons test or one-way ANOVA, based on the normality of data). All comparisons were performed, but only those with statistical significance are annotated. Exact p-values are indicated in the figure (ns = not significant).

Since this model forms MAC upon sera exposure,^19^ transcriptional changes in expression of complement components were assessed. Notably, α-subunit-specific mAb significantly increased expression of early classical pathway components *C1S* (C1s), *C3AR1* (C3aR), *C5* (C5), and terminal MAC component *C9* (C9), as well as complement regulators *CD55* (CD55) and *CD59* (CD59) (Fig. 6b, c, e, f, j, k; n = 12; *C1S*, control: mean ± SEM = 1.3 ± 0.2, 01b (α) mAb: mean ± SEM = 11.3 ± 3.7, 09 (β) mAb: mean ± SEM = 3.2 ± 1.4; *C3AR*, control: mean ± SEM = 2.2 ± 1.0, 01b (α) mAb: mean ± SEM = 19.2 ± 6.6, 09 (β) mAb: mean ± SEM = 4.2 ± 1.6; *C5*, control: mean ± SEM = 1.6 ± 0.4, 01b (α) mAb: mean ± SEM = 5.2 ± 1.1, 09 (β) mAb: mean ± SEM = 3.3 ± 0.9; *C9*, control: mean ± SEM = 1.6 ± 0.5, 01b (α) mAb: mean ± SEM = 13.8 ± 4.0, 09 (β) mAb: mean ± SEM = 3.3 ± 1.1; *CD55*, control: mean ± SEM = 1.4 ± 0.4, 01b (α) mAb: mean ± SEM = 7.9 ± 3.4, 09 (β) mAb: mean ± SEM = 4.5 ± 1.7; *CD59*, control: mean ± SEM = 1.2 ± 0.2, 01b (α) mAb: mean ± SEM = 12.4 ± 5.7, 09 (β) mAb: mean ± SEM = 2.7 ± 0.6; Kruskal–Wallis test, p-value = 0.018, 0.03, 0.011, 0.019, 0.013 and 0.0072, respectively). Additionally, *C4A* (C4), *CFB* (Factor B), *CFH* (Factor H) and *CFI* (Factor I) showed trends of increase (Fig. 6d, g–i). β-subunit-specific mAb similarly upregulated some complement components, particularly *C1R* (C1r) (Fig. 6a, n = 12, control: mean ± SEM = 1.1 ± 0.2, 01b (α) mAb: mean ± SEM = 1.7 ± 0.5, 09 (β) mAb: mean ± SEM = 2.8 ± 0.6; one-way ANOVA, p-value = 0.05), but these effects were less pronounced than with α-subunit-specific mAb. These results reveal α-subunit-specific mAb-mediated inhibition upregulates several complement components, suggesting a potential contribution towards enhanced MAC formation.

### C3 inhibition ameliorates α-mAb-mediated pathogenic effects

**Fig. 7:**
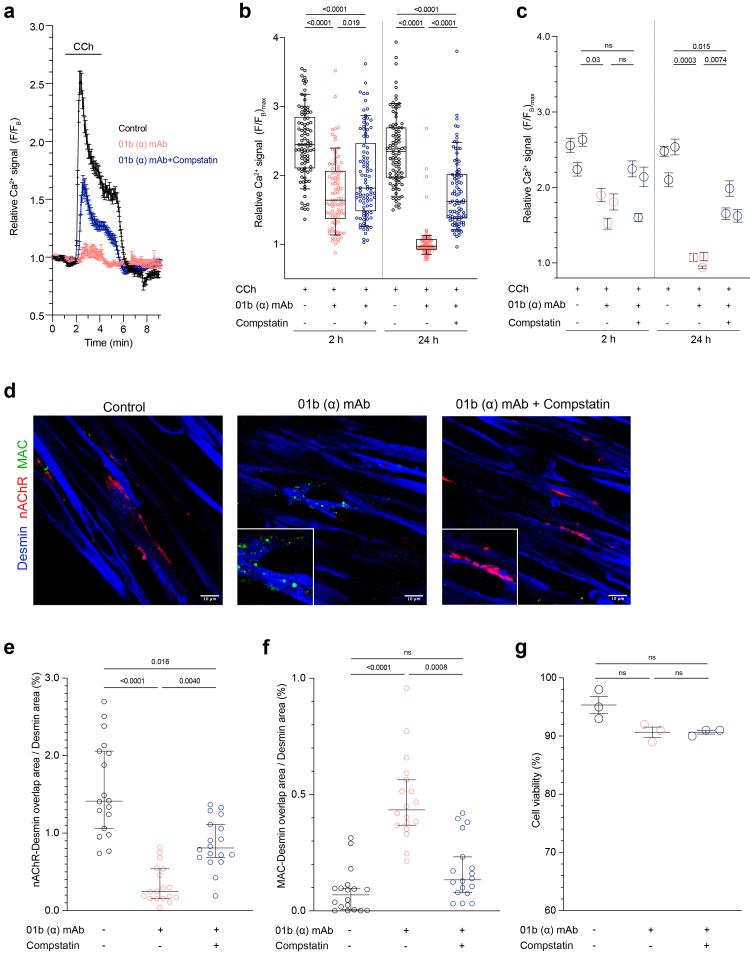
**C3-binding peptide compstatin ameliorates inhibitory effects of α-subunit-specific mAb on cholinergic calcium signaling, AChR distribution and MAC formation. (a)** Representative traces of CCh-induced calcium responses from control myotubes or α-subunit-specific mAb treated myotubes or myotubes simultaneously treated with α-subunit-specific mAb and C3-binding peptide compstatin (100 μM) for 24 h. Application of CCh is indicated by horizontal line. Data are presented as mean ± SEM of relative intensity $\left(F/F_{B}\right)$ from 30 individual myotubes in each group. **(b)** Quantification of maximal CCh-induced calcium responses ((F/F_B_)_max_) for the myotubes as shown in (a) for 2 h and 24 h time points. Dataset shown represents **(b)** individual myotubes (n = 3 experiments, a total of 90 per group; box-and-whisker plots with median, interquartile range, and 10th–90th percentiles; Kruskal–Wallis test) and **(c)** average values (n = 3 experiments; mean ± SEM; one-way ANOVA). Statistical analyses were performed separately for the 2 h and 24 h time points. **(d)** Representative immunofluorescence images of myotubes stained for desmin (green), nAChR (red), and membrane attack complex (MAC, magenta) under control conditions, α-subunit-specific mAb treatment, or mAb with compstatin. Insets show magnified regions highlighting nAChR distribution and MAC deposition. Scale bar, 10 μm. Quantification of **(e)** nAChR-desmin overlap area and **(f)** MAC-desmin overlap area, each normalised to total desmin area (%). Data were obtained from 18 images per group from three independent experiments (n = 3 experiments; median with 95% confidence intervals; Kruskal–Wallis test followed by Dunn’s multiple comparisons test). **(g)** Cell viability was assessed by the trypan blue exclusion method after 24 h of treatment under control conditions, α-subunit-specific mAb, or mAb with compstatin. Data are presented as percentage of viable cells (n = 3 experiments; mean ± SEM; Kruskal–Wallis test followed by Dunn’s multiple comparisons test). Exact p-values are indicated in the figure (ns = not significant).

**Fig. 8:**
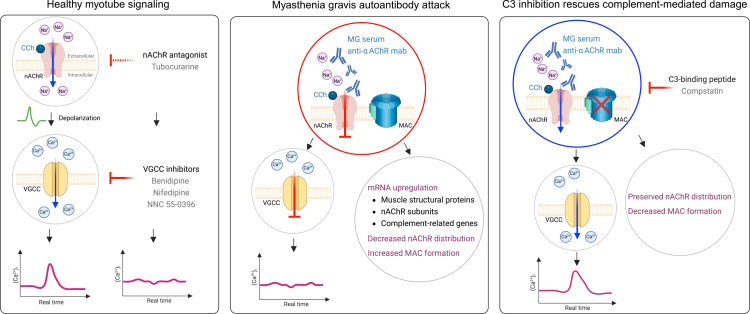
**Schematic of hampered cholinergic calcium signaling by MG pathogenic antibodies, and reconstitution by complement C3 inhibition in human myotubes.** In healthy human myotubes (left panel), the cholinergic agonist carbamoylcholine chloride (CCh) binds to nAChRs induces depolarisation and calcium responses, which can be blocked by pharmacological inhibition of nicotinic acetylcholine receptors (nAChRs) and voltage-gated calcium channels (VGCCs), demonstrating functional coupling between them. Autoimmune attack by pathogenic antibodies in myasthenia gravis (MG) serum or by recombinant monoclonal antibodies (mAbs) leads to transcriptional alterations, reduced nAChR distribution, formation of the complement membrane attack complex (MAC), and impaired choline-induced calcium responses (middle panel). Inhibition of complement activation by a C3-binding peptide rescues the calcium signal, reduces MAC formation, and preserves nAChR distribution suppressed by anti-α nAChR mAb (right panel). This figure was created with BioRender.

Since inhibition of myotubes with α-subunit-specific mAb upregulated early classical complement pathway components, we investigated whether pharmacological inhibition of complement activation could rescue the impaired cholinergic signaling. Compstatin, a C3-binding peptide that prevents C3 cleavage and downstream MAC formation,^31^^,^^32^ was co-applied with α-subunit-specific mAb. Based on previous findings that MAC formation occurs within 2 h of MG sera exposure,^19^ calcium responses were assessed after both short-term (2 h) and prolonged (24 h) treatments. Strikingly, after 24 h exposure, compstatin significantly reversed the complete inhibition of cholinergic calcium signals caused by α-subunit-specific mAb (Fig. 7a–c; Fig. 7b, n = 3; control: median = 2.3, 10th–90th percentiles = 1.7–3.0; 01b (α) mAb: median = 1.0, 10th–90th percentiles = 0.9–1.1, (α) mAb + compstatin: b: median = 1.6, 10th–90th percentiles = 1.2–2.5; Kruskal–Wallis test, p-value < 0.0001; Fig. 7c, n = 3; control: mean ± SEM = 2.3 ± 0.1; 01b (α) mAb: mean ± SEM = 1.0 ± 0.04, 01b (α) mAb + compstatin: mean ± SEM = 1.7 ± 0.1; one-way ANOVA, p-value = 0.0074) whereas it partially restored the responses after 2 h treatment (Fig. 7b and c, n = 3; control: median = 2.4, 10th–90th percentiles = 1.8–3.2; 01b (α) mAb: median = 1.6, 10th–90th percentiles = 1.1–2.4, (α) mAb + compstatin: median = 1.8, 10th–90th percentiles = 1.3–2.9; Kruskal–Wallis test, p-value < 0.0001; n = 3, control: mean ± SEM = 2.5 ± 0.1; 01b (α) mAb: mean ± SEM = 1.7 ± 0.1, 01b (α) mAb + compstatin: mean ± SEM = 2.0 ± 0.1; one-way ANOVA, p-value = 0.031). To determine whether the observed impairment in cholinergic calcium signaling is associated with altered nAChR distribution and terminal MAC formation, immunocytochemistry was performed. Treatment with the α-subunit-specific mAb reduced the nAChR, indicating loss of receptors from myotubes (Fig. 7d and e; Fig. 7e, n = 3, control: median [95% confidence interval (CI)] = 1.6 [1.0; 2.0]; 01b (α) mAb: median [95% CI] = 0.3 [0.2; 0.5]; 01b (α) mAb + compstatin: median [95% CI] = 0.9 [0.7; 1.1]; Kruskal–Wallis test, p-value < 0.0001). In parallel, increased MAC deposition on myotubes was detected (Fig. 7d and f; Fig. 7f, n = 3, control: median [95% (CI)] = 0.08 [0.01; 0.1]; 01b (α) mAb: median [95% CI] = 0.4 [0.4; 0.6]; 01b (α) mAb + compstatin: median [95% CI] = 0.1 [0.07; 0.2]; Kruskal–Wallis test, p-value < 0.0001). Compstatin treatment partially restored nAChR localisation on myotubes and reduced MAC deposition (Fig. 7d–f; Kruskal–Wallis test, p-value = 0.004 and 0.0008, respectively). Importantly, no significant change in cell viability was observed after 24 h exposure to the α-subunit-specific mAb compared to untreated cells (Fig. 7g), indicating that the impairment in calcium signaling reflects complement-associated sub-lytic functional effects rather than loss of cell membrane integrity. These results support the therapeutic potential of C3-targeted complement inhibition in preserving neuromuscular transmission (Fig. 8).

## Discussion

The primary aim of this study was to evaluate the inhibitory effects of nAChR-targeting antibodies, specifically, patient-derived polyclonal antibodies and recombinant pure mAbs, on cholinergic signaling cascades and related cellular mechanisms in a human muscle cell model of MG.^19^ Results confirm robust, dose-dependent cholinergic calcium responses and membrane depolarisation via functional nAChRs and VGCCs in human myotubes, thereby demonstrating their tight coupling. This model recapitulated key pathological features of MG, including nAChR loss and functional impairment, as well as complement component upregulation and MAC formation. The impaired cholinergic calcium responses caused by α-subunit-specific mAb were associated with reduced nAChR levels and increased MAC deposition on myotubes, indicating that complement-mediated loss of receptor availability leads to functional disruption. Importantly, pharmacological inhibition of complement activation with a C3-binding peptide (compstatin) restored the cholinergic calcium signal suppressed by α-subunit-specific mAb, reduced MAC deposition, and preserved nAChR distribution, suggesting a prominent role of complement C3 in antibody-driven neuromuscular dysfunction.

Mechanistically, this HSMM-derived myotube model supports in vivo muscle physiology, in which nAChR activation depolarises the membrane, thereby opening VGCCs and driving calcium influx. The complete abolition of calcium responses by the broad-spectrum VGCC blocker benidipine, together with the differential effects of selective inhibitors, nifedipine (high-voltage-activated L-type channels), which caused a partial but significant reduction (18.4% inhibition), and NNC 55-0396 (low-voltage-activated T-type), which produced a stronger suppression (59.0% inhibition), indicates coordinated involvement of both L-type and T-type VGCCs in the observed signals, with a predominant contribution from T-type channels. Transcript profiling identified *Cacna1S* (Ca_V_1.1; L-type) and *Cacna1H* (Ca_V_3.2; T-type), with *Cacna1H* being higher in this model. The nAChR activation generated a small membrane depolarisation, consistent with the notion that it will activate Ca_V_3.2 channels (low-voltage-activated VGCCs). Ca_V_3.2 channels modulate intracellular calcium dynamics during late myotube formation and support calcium homeostasis.^33^ Inhibition of T-type channels has been reported to disrupt calcium homeostasis and trigger endoplasmic reticulum stress in C2C12 cells and to alter muscle fiber size in mice,^11^ highlighting their importance for muscle cell integrity. Conversely, Ca_V_1.1, exclusively expressed in skeletal muscles, serves as the voltage sensor for ECC^34^ and mediates a small but functionally relevant calcium influx to sustain muscle function.^35^ Together, these findings point to a physiologically coherent pathway coupling nAChR and VGCCs through depolarisation that is conserved in these myotubes.

We observed higher expression of Ca_V_3.2 than Ca_V_1.1 in human myotubes, but in the rodent models, functionality and abundance of Ca_V_3.2 channels are not clear. In neonatal mouse skeletal muscles, T-type calcium currents decline progressively with postnatal maturation, but reappeared under denervation in the culture condition.^36^ Another study reported an increase in Ca_V_3.2 positive myofibers from postnatal day 5–10.^37^ Further, agrin-mediated signaling in C2C12 cells links LRP4 and Ca_V_3.2 expression.^37^ Thus, Ca_V_3.2 expression in our differentiated human myotubes maybe associated with agrin-driven NMJ-like signaling state.

Our finding that MG sera has inhibitory effects on cholinergic calcium signals in human myotubes is consistent with prior patch-clamp electrophysiological studies assessing nAChR channel currents in mouse myotubes^15^^,^^16^ and in a human rhabdomyosarcoma cell line.^17^ Unlike previous studies, which applied sera acutely (20 s–2 min) to demonstrate direct inhibition, we employed prolonged incubation to better mimic the chronic nature of MG in vivo. Earlier cell-based assays and transcriptomic studies have shown that AChR + MG sera can induce nAChR internalisation and degradation in muscle cells,^8^^,^^38^, ^39^, ^40^ mechanisms that likely contribute to the suppression observed in our study. Whereas patch-clamp techniques assess either whole-cell channel activity from a single cell or single-channel current from a patch on a single cell, real-time calcium imaging enables noninvasive, simultaneous monitoring of multiple myotubes, providing a broader physiological perspective.

In this study, in addition to MG sera, we used recombinant, purified antibodies against the α- and β-subunits, which enabled dissection of subunit-specific functional effects, highlighting the dominant contribution of the α-subunit to receptor signaling. In muscle-type nAChRs, ligand binding occurs at the interfaces between α1 and adjacent ε/γ and δ subunits,^3^ with the α subunits mediating the conformational changes required for channel opening.^41^ Moreover, the MIR resides at the extreme top end of the α subunits, rendering it highly accessible to circulating antibodies.^42^ In contrast, the β-subunit contributes to nAChR clustering via MuSK-mediated phosphorylation,^43^ a role that may be less involved in immediate channel gating. Consistent with these mechanistic distinctions, antibodies targeting the α-subunit are more effective than those targeting β in inducing severe MG symptoms in the experimental autoimmune MG (EAMG) model^44^ and in promoting nAChR cross-linking in human rhabdomyosarcoma cells.^45^ Furthermore, real-time physiological analysis of α- and β-subunit blockade in a live-cell system reinforces the mechanistic link between pathogenic antibody binding sites and receptor function. While previous studies have primarily focused on detecting subunit-specific antibodies in peripheral blood, our findings demonstrate that blockade of the α-subunit can directly impair nAChR-mediated signaling in human myotubes in vitro.

Recent studies demonstrate that individual mAbs, including those targeting the α-subunit, can exert multiple pathogenic mechanisms simultaneously.^18^ Accordingly, receptor blockade and antigenic modulation are likely to contribute to the short-term (2 h) impairment of cholinergic calcium responses induced by the α-subunit-specific mAb. In contrast, the pronounced effects after prolonged exposure (24 h), together with increased MAC deposition, reduced nAChR levels, and partial reversal of these changes with complement inhibition, supporting a prominent contribution of complement-mediated functional impairment without affecting cell viability. Moreover, previous studies have shown that synergistic interactions between antibodies targeting different nAChR subunits can enhance complement activation.^46^ Future studies combining antibodies against multiple nAChR subunits may help determine whether such synergy exists and further amplifies functional impairment in this model.

Antigen- and subunit-specific insights hold strong translational potential for targeted therapeutic strategies, including precision immunotherapy. For instance, an α1-subunit recombinant mutant peptide (α1-ECD_m_), encompassing the majority of auto-epitopes in MG, has effectively suppressed EAMG,^47^ suggesting the feasibility of selectively modulating autoimmune responses without inducing broad immunosuppression. Furthermore, large-scale serologic analyses in patients with AChR + MG have linked the dominance of α-subunit-specific autoantibodies to late-onset MG with lower antibody titers, whereas the dominance of γ -subunit-specific autoantibodies were associated with early-onset MG and higher antibody titers.^48^ Here, we demonstrate that complement inhibition restores α-subunit-specific mAb-mediated suppression of nAChR function, underscoring the rationale for complement-directed therapies to preserve neuromuscular transmission. Stratification based on antigen and subunit specificity could guide tailored interventions for future immunotherapies.

Additionally, functional blockade of nAChRs with MG sera and/or subunit-specific mAb in myotubes led to transcriptional upregulation of α-actin and desmin (muscle specific proteins related to contractile machinery), complement components C1s, C3aR, C5, and C9, and also an observed trend of enhanced expression of nAChR and VGCC subunits. Increased α1-subunit mRNA has been reported in EAMG^49^^,^^50^ and muscle biopsies from patients with MG,^51^ but it did not functionally restore receptor loss in EAMG,^50^ suggesting a non-functional feedback loop. The β- and δ-subunit transcripts were increased in muscle biopsies from patients with MG and in rhabdomyosarcoma cells treated with nAChR mAbs, consistent with our findings.^51^ Given that the γ subunit forms a high-affinity agonist binding site on nAChRs,^52^ it’s upregulation may serve as a compensatory attempt to regain cholinergic function under receptor blockade. Inhibition of membrane depolarisation increases AChE transcription and secretion in muscle fibers,^53^ reflecting AChE transcript upregulation with MG sera and α-subunit mAb treatment, which can be a compensatory mechanism. VGCC transcripts (*Cacna1S* and *Cacna1C*) showed upward trends in response to MG sera, potentially supporting calcium homeostasis and muscle function under impaired nAChR signaling. Increased α-actin and desmin transcriptional levels are consistent with previous findings that recombinant AChR mAbs alter actin family gene expression in human muscle cells^40^ and that actin polymerisation is required for nAChR internalisation.^54^ Elevated levels of C5 transcript have previously been reported in AChR + MG sera,^19^ and we observed corresponding mRNA upregulation in myotubes in this study. Complement regulator proteins CD55, which restrict convertase formation, and CD59, which inhibits MAC assembly,^55^^,^^56^ were elevated in muscle tissue from patients with MG.^57^ This increase corresponds to the mAb-mediated upregulation observed in our study, suggesting a potential intrinsic protective mechanism against complement-driven damage. Whether these transcriptional alterations are a consequence of functional ablation of nAChRs or occur in parallel to enhance receptor dysfunction requires further investigation of protein levels using functional rescue experiments. These findings together indicate that complement activation, downstream of antibody binding, can also contribute to nAChR dysfunction.

Current approved complement-targeting therapies for MG, including eculizumab, ravulizumab, and zilucoplan, focus on inhibiting C5 to block terminal complement activation.^58^ In contrast, our study demonstrates that proximal inhibition of complement activation at the level of C3 restores cholinergic calcium responses and preserves nAChR distribution disrupted by the α-subunit-specific mAb. This observation aligns with findings from a preprint regarding passive transfer MG (PTMG) models, where C3 knockdown by siRNA reduced nAChR loss, decreased complement deposition, and improved muscle function.^59^ Because C3 represents the central convergence point of all complement pathways, classical, alternative, and lectin, blocking C3 can provide broader protection than C5 blockade. Moreover, targeting C3 not only prevents MAC-mediated damage but also blocks the generation of inflammatory fragments such as C3a, which is higher in AChR + MG^19^^,^^60^ and associated with inflammatory signaling.^61^ Together, these results highlight C3 as a promising upstream therapeutic target that could complement, or even surpass, current C5-directed therapeutic strategies.

While this study provides mechanistic insights into cholinergic signaling and the effects of MG-associated antibodies, several limitations should be acknowledged. The cultured human myotubes may exhibit a mixed fetal and adult nAChR subunit composition, as the developmental γ-to-ε subunit switch is regulated by motor neuron innervation and electrical activity^62^; therefore, this model may not fully reflect mature myotubes in vivo. In addition, the responsiveness of myotubes may vary slightly with cellular age, and fluorescent probes for calcium imaging and membrane potential measurements are susceptible to photobleaching, which may contribute to minor variability in choline-induced maximal calcium responses. Moreover, the small number of patient sera may limit generalisability, and the use of sera from a single clinical site further reduces sample diversity. The potency of mAbs may contribute to the markedly observed functional and transcriptional effects, compared with MG sera with milder effects. Additionally, mAbs can vary in potency, thus future studies using different concentrations of mAbs could be elucidative. Furthermore, our interventions were primarily pharmacological and antibody-based; future studies employing genetic approaches, such as siRNA, antisense oligonucleotide, or CRISPR-mediated knockdown/out, could strengthen causal inference to expand mechanistic understanding. Finally, the C3 complement inhibition may have other consequences such as anti-inflammatory effects, apart from the major effect on MAC inhibition.

In conclusion, our study provides three mechanistic insights into MG pathophysiology and novel druggable targets. First, we demonstrate that nAChR-mediated depolarisation activates VGCCs, with T-type Ca_V_3.2 channels prominently contributing to calcium influx. Second, antibody targeting the AChR α-subunit completely abolishes cholinergic calcium signaling, reduces nAChR levels, and induces MAC formation, underscoring the α-subunit’s dominant role in receptor activation and in the autoimmune attack. Third, MG-associated antibodies also upregulate complement components, and inhibition of C3 effectively preserves nAChR distribution, reduces MAC formation, and restores nAChR-dependent calcium responses. These findings highlight the α-subunit as a critical pathogenic target and identify C3 as a promising upstream therapeutic node, supporting the development of subunit-specific interventions and proximal complement inhibition as precision strategies to preserve neuromuscular transmission and improve clinical outcomes in MG.

## Contributors

**Yu-Fang Huang**: Conceptualisation, Data curation, Formal analysis, Investigation, Methodology, Visualisation, Writing—original draft, Writing—review and editing.

**Amol K. Bhandage**: Conceptualisation, Data curation, Formal analysis, Investigation, Methodology, Visualisation, Supervision, Writing—review and editing.

**Miriam Fichtner:** Methodology, Resources, Writing—review and editing.

**Anna Rostedt Punga**: Conceptualisation, Investigation, Funding acquisition, Project administration, Resources, Supervision, Writing—review and editing.

All authors have read and approved the final version of the manuscript. Yu-Fang Huang, Amol K. Bhandage, and Anna Rostedt Punga verified the underlying data.

## Data sharing statement

The data and materials supporting the conclusions of this article are included in this published article (and its Supplementary information files). Related sample metadata are available from the corresponding author on request. Still, individual-level data can only be released under a suitable data-sharing agreement, owing to restrictions on informed consent.

## Declaration of interests

ARP has received speaking honoraria from UCB and Alexion; consulting fees from Dianthus, Merck, and Toleranzia; and has served on Data Safety Monitoring Boards or Advisory Boards for Novartis and Argenx, all outside the submitted work. MLF has received speaker honoraria from Alexion Pharmaceuticals and is a member of the Alexion Academy in Germany. Additionally, MLF has received financial research support from argenx. MLF is an active member of both the German Myasthenia Gravis Society and the German Association for Patients Affected by Muscle Diseases. In relation to this study, the authors have no competing interests.

## Appendix A Supplementary data

Supplementary video

Certificate of AnalysisSkeletal Muscle Myoblasts

EBIOM-D-25-06733 full blots
