# Supplementary material for: Complement C3 inhibition restores myasthenia gravis AChR antibody-mediated muscle pathophysiology
Source: eBioMedicine. 2026 Jun 8;129:106322. doi: 10.1016/j.ebiom.2026.106322 (PMC13264364; doi:10.1016/j.ebiom.2026.106322)
Supplement: Supplementary Table S1 [file mmc1.docx]

**Supplementary Table. 1. Cohort demographics of myasthenia gravis (MG) patients and healthy controls (HCs)**

|  | Sex | Age at sample collection | AChR antibody titer (nM) | Clinical subtype | MG-ADL | Disease duration (yrs) | Medications |
| --- | --- | --- | --- | --- | --- | --- | --- |
| MG 1 | F | 18 | 0.3 | Generalized | 12 | 7 | Mestinon, Prednisone, Ventolin |
| MG 2 | F | 45 | 6.7 | Generalized | 11 | 6 | Prednisone, Mestinon, Methotrexat, Rituximab |
| MG 3 | F | 38 | 2.3 | Generalized | 7 | 3 | Prednisone, Mestinon, Rituximab |
| HC 1 | F | 18 | - | - | - | - | - |
| HC 2 | F | 45 | - | - | - | - | - |
| HC 3 | F | 38 | - | - | - | - | - |

Abbreviations: F, female; AChR, acetylcholine receptor; MG-ADL, Myasthenia Gravis Activities of Daily Living.
