# Supplementary material for: Complement C3 inhibition restores myasthenia gravis AChR antibody-mediated muscle pathophysiology
Source: eBioMedicine. 2026 Jun 8;129:106322. doi: 10.1016/j.ebiom.2026.106322 (PMC13264364; doi:10.1016/j.ebiom.2026.106322)
Supplement: Supplementary Table S2 [file mmc2.docx]

**Supplementary Table. 2. Primer list**

| **Protein (Gene)** | **Forward Primer** | **Reverse Primer** | **Amplicon size (bp)** |
| --- | --- | --- | --- |
| **Complement components** |  |  |  |
| C1r (C1R) | TCACAGTCCCCACGGGATAC | CCAGTGGAGAACCCAGTTGC | 147 |
| C1s (C1S) | GAATTGCGGAGTTAATTGCAG | TATGGTTTGGGATAATTGGGAC | 81 |
| C3a receptor (C3AR1) | GAAGTTTAGCAATGGCGTC | GGCTGTGAGAGTAGGTCAG | 61 |
| C4 (C4A) | TGGACATGAAGAACACGAC | TTGCTTCCATCGTGTACTC | 81 |
| C5 (C5) | TGAACTCTTTGAAGTTGGGT | ATGGTACACTGTTTATCTGGTC | 81 |
| C9 (C9) | ACGACCAGTTATGACCCAG | TGTGACCATTCACTCCAGG | 89 |
| Factor B (CFB) | TGTCATTGATGAGATCCGGG | ACATAGACATCCAGATAATCCTCC | 81 |
| Factor H (CFH) | CGAGATGTACCTTGAAACCT | AGTATGGTCTACGCATATTCTC | 81 |
| Factor I (CFI) | ACTGTGTTGTAAAGCATGCC | CCATTGCATTGATACTGGCT | 81 |
| CD59 (CD59) | CTGCCATTCAGGTCATAGC | GATGAACAATTGACGGCTG | 81 |
| CD55 (CD55) | GGTCAGATTGATGTACCAGG | GCCAAATAATTTGTACCCTGTG | 81 |
| **VGCC subunits** |  |  |  |
| Ca_V_ 1.1 (Cacna1S ) | TGTCATCCTCAGTGAGATCG | TCATCTGGGTCAACGTTCC | 90 |
| Ca_V_ 1.2 (Cacna1C) | TTCCAACCTGGAACGAGTGG | AGGCATTGGGGTGAAAGAGG | 101 |
| Ca_V_ 1.3 (Cacna1D) | GGTGATCCCCTTCCCCATTC | ATAGTTTGCCTCGTTCGCGT | 162 |
| Ca_V_ 1.4 (Cacna1F ) | CCTGGGATCCGACATGGAAG | ACTCAGTCTGGTTCAGCGTG | 89 |
| Ca_V_ 2.1 (Cacna1A ) | TTCAACATCGTCTTCACCTC | GCGGAAATAATTCAGAATCCC | 81 |
| Ca_V_ 2.2 (Cacna1B ) | GGAATGTCTTTGACTTTGTCAC | TGAAATTGTTCGTTTCCGC | 81 |
| Ca_V_ 2.3 (Cacna1E ) | GATGGGACTCCTTCGGCAAA | CCCGTCAGGATCTGGAACAC | 71 |
| Ca_V_ 3.1 (Cacna1G ) | CCACGTGGTCCTTGTCATCA | GGGTCAGGAAGATGCGTTCA | 98 |
| Ca_V_ 3.2 (Cacna1H) | CATCTTCCAGGTGATCACG | ATGAAGTTGTAGAATGAGTGGG | 81 |
| Ca_V_ 3.3 (Cacna1I ) | GTGGAGATCATGTACTACGTG | GGAGCCCACTATGATAAGC | 81 |
| **nAChR subunits** |  |  |  |
| α1 (CHRNA1) | CCGAGGTGAAAAGTGCCATCGA | TCCGAGGAGTATGTGGTCCATC | 128 |
| α2 (CHRNA2) | GGTCTGAGGATGCTGACTCTTC | TAGGAACGGAGGCAGAAAGAGG | 134 |
| α3 (CHRNA3) | TGGAGACCAACCTGTGGCTCAA | CAGCACAATGTCTGGCTTCCAG | 128 |
| α4 (CHRNA4) | CGTCCAGTACATTGCAGACCAC | TCCAGAGGAAGATGCGGTCGAT | 104 |
| α5 (CHRNA5) | CTTGCAATATCTCAATTGGTGG | TATCCATTCCTGTTTCAACCAG | 81 |
| α6 (CHRNA6) | GGCATTGAGACTCTTCGCGTTC | GCTGGTGGAGTCCAGGTTATCA | 146 |
| α7 (CHRNA7) | TGGTGACAGTGATCGTGCTGCA | GCCTCTTCATTCGCAGGAACCA | 117 |
| α9 (CHRNA9) | CTAATGCTCTTCGTCCAGTGGAA | GTGAGATAGGCATCGTGCCAGA | 151 |
| α10 (CHRNA10) | ATGCCTACCTACGATGGGA | GCGTCGGCTTTGTTATAGAG | 109 |
| β1 (CHRNB1) | AGTGTCGTGGTTCTCAACCTGC | TTAGACGCAGGTACAGCGGAAG | 106 |
| β2 (CHRNB2) | CTCCCTTCCAAACACATCTGGC | GATGCTGCCATCATAGGAGACC | 111 |
| β3 (CHRNB3) | GACAGACCACAAGTTACGCTGG | TGGTCATCAGGGAGCCTTCGAA | 140 |
| β4 (CHRNB4) | CATCTCCATCAAGCTGCAG | AGTCCATTCCTGTTTCAGC | 100 |
| δ (CHRND) | TGGCTCAGTCTGTCTTCCTGCT | GAGCACGATGACACAGATCACC | 137 |
| γ (CHRNG) | CACCAACCTCATCTCCCTGA | GAGGGCCACCTCGAAGACAC | 202 |
| ε (CHRNE) | CTGCTCCTCTTGGGGCTTCTC | CTTGAGGCTGATGGTGACAGTAT | 138 |
| **Muscle markers** |  |  |  |
| a-actin (ACTA1) | CACGATGTACCCTGGGATCG | GCCGATCCACACCGAGTATT | 118 |
| Desmin (DES) | CATGAAGAGGAGATCCGTG | GTCTGGCTTAGACATGTCC | 81 |
| Acetylcholinesterase (ACHE) | GTTCTCCTTCGTGCCTGTGGTA | ATACGAGCCCTCATCCTTCACC | 124 |
