# Supplementary material for: Complement C3 inhibition restores myasthenia gravis AChR antibody-mediated muscle pathophysiology
Source: eBioMedicine. 2026 Jun 8;129:106322. doi: 10.1016/j.ebiom.2026.106322 (PMC13264364; doi:10.1016/j.ebiom.2026.106322)
Supplement: Supplementary Table S3 [file mmc3.docx]

**Supplementary Table. 3. Antibody list**

| **Antibody** | **RRID** | **Catalog number** | **Manufacturer** |
| --- | --- | --- | --- |
| Nicotinic Acetylcholine R alpha 1/CHRNA1 Antibody (mAb 192) | AB_3411441 | NBP2-81070 | Novus Biologicals |
| Rabbit anti-Rat IgG (H+L) Secondary Antibody, Biotin | AB_228448 | 31834 | Thermo Fisher Scientific |
| Anti-Desmin antibody [Y66] - Cytoskeleton Marker | AB_731901 | ab32362 | Abcam |
| Anti-Sarcomeric Alpha Actinin antibody [EA-53] | AB_307264 | ab9465 | Abcam |
| Anti-Complement component C5b-9 (human) | AB_1072776 | DIA 011-01-02 | BioPorto |
| Donkey Anti-Rabbit IgG H&L (Alexa Fluor® 405) | AB_2715515 | ab175649 | Abcam |
| Goat Anti-Mouse IgG H&L (Alexa Fluor® 488) | AB_2576208 | ab150113 | Abcam |
