# Supplementary figures and images for: Complement C3 inhibition restores myasthenia gravis AChR antibody-mediated muscle pathophysiology
Source: eBioMedicine. 2026 Jun 8;129:106322. doi: 10.1016/j.ebiom.2026.106322 (PMC13264364; doi:10.1016/j.ebiom.2026.106322)

**a**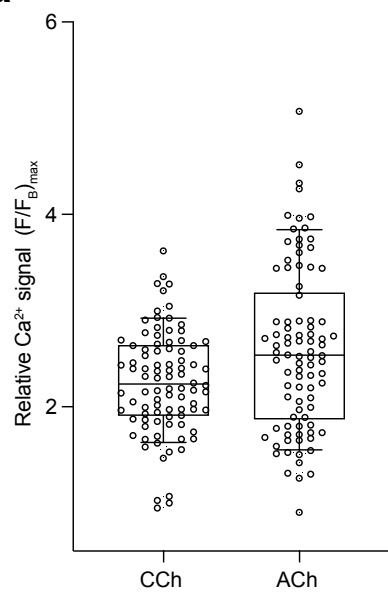**b**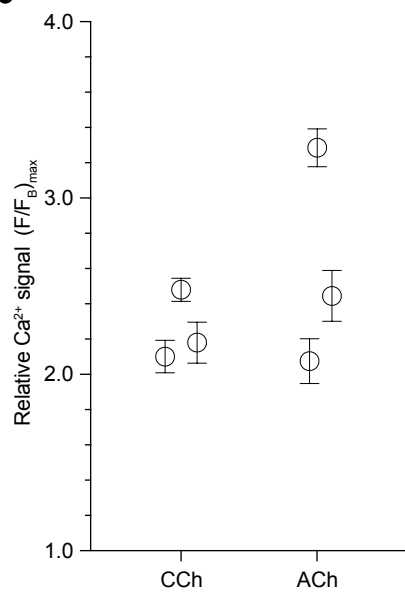

Supplement: Supplementary Fig. S1 [file mmc4.pdf]

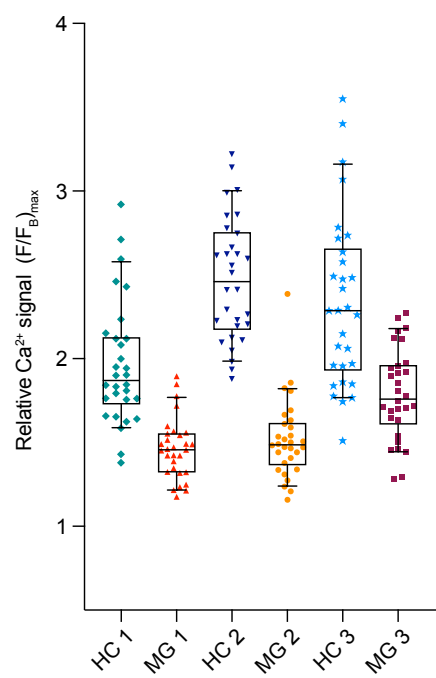

Supplement: Supplementary Fig. S2 [file mmc5.pdf]

figure 1c

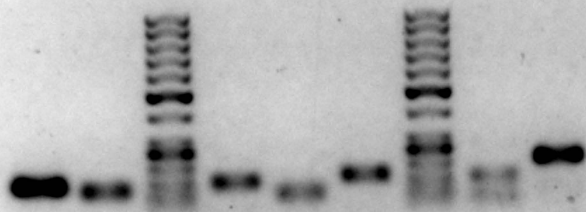

figure 1d

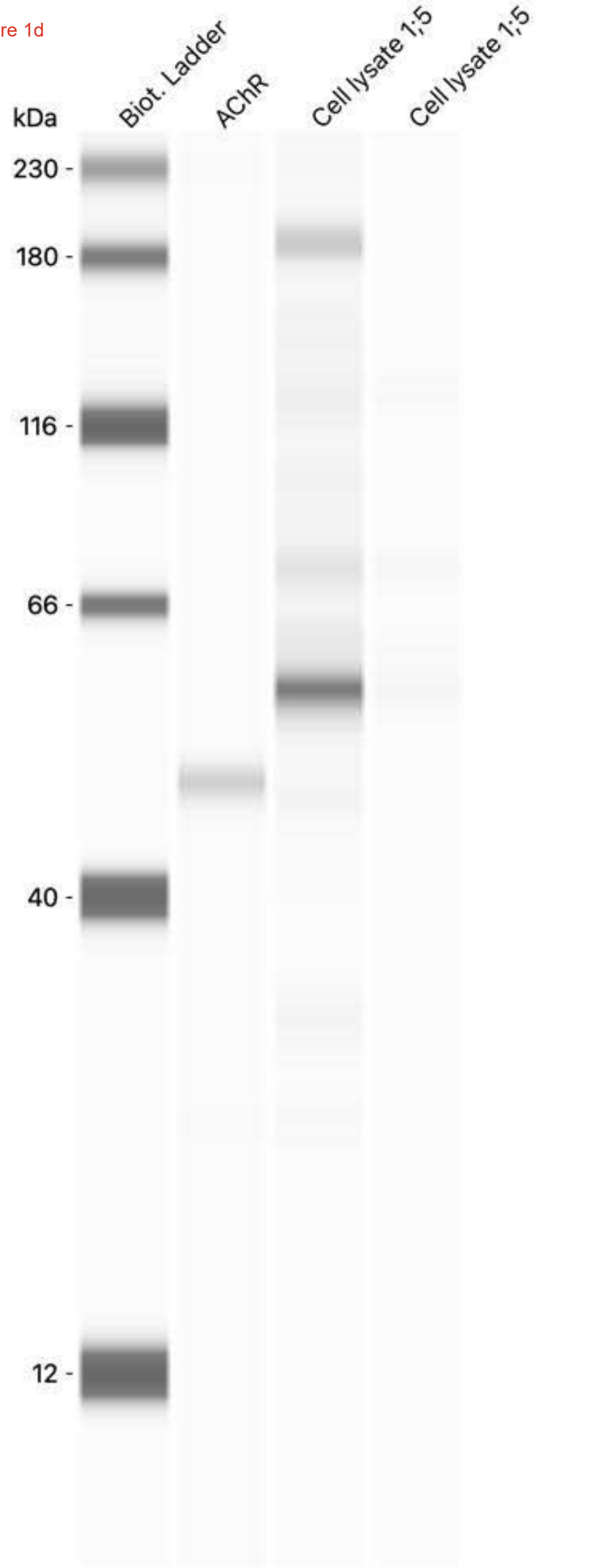

figure 3f

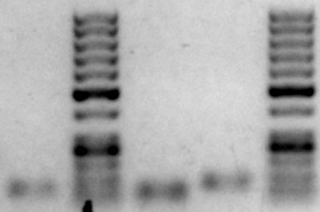

Supplement: EBIOM-D-25-06733 full blots [file mmc8.pdf]
