# Supplementary material for: Complement C3 inhibition restores myasthenia gravis AChR antibody-mediated muscle pathophysiology
Source: eBioMedicine. 2026 Jun 8;129:106322. doi: 10.1016/j.ebiom.2026.106322 (PMC13264364; doi:10.1016/j.ebiom.2026.106322)
Supplement: Certificate of AnalysisSkeletal Muscle Myoblasts [file mmc7.pdf]

Lonza

Certificate of Analysis

eShop Dummy Customer SE
used in eShop only
SE-

Print Date: 27-Apr-2026

Product Name: HSMM-Muscle Myoblasts SkGM-2, cryo amp
Material Number: CC-2580
Batch No: 21TL138913
Manufacturing Date: 27-Jun-2021

Test	*RESULT*	*SPECIFICATION MIN*	*MAX*	*UNIT*
Tissue Acquisition Number	43293			
DONOR CHARACTERISTICS				
Age	31			
Race	C			
Sex	FEMALE			
VIRUS TESTING				
HIV Test	Not Detected			
HBV Test	Not Detected			
HCV Test	Not Detected			
MICROBIAL TESTING				
Sterility Test	Negative	***	***	
Mycoplasma	Negative	***	***	
CELL PERFORMANCE TESTING				
Cell Passage Frozen	2'			
Viability	97	>=70%	***	%
Cell Count (Cells/ml)	1080000	>=500,000	***	
Seeding Efficiency	60	>=50%	***	%
Doubling Time (hours)	28	12	36	hrs
Desmin for myogenicity (differentiation)	Pass	Pass: >=60% Positive	***	

This lot has been reviewed by Quality Assurance in compliance with requirements of Lonza's Quality System. This document was generated from a validated Part 11-compliant electronic system and thus handwritten signatures are not required.

Lonza Cologne GmbH
Nattermannallee 1
D-50829 Cologne

For Technical Assistance, call 1-800-521-0390

Lonza

Certificate of Analysis

eShop Dummy Customer SE
used in eShop only
SE-

Print Date: 27-Apr-2026

Product Name: HSMM-Muscle Myoblasts SkGM-2, cryo amp
Material Number: CC-2580
Batch No: 21TL138913
Manufacturing Date: 27-Jun-2021

Test	*RESULT*	*SPECIFICATION MIN*	*MAX*	*UNIT*
HLA A Set 1	01:01:01G			
HLA A Set 2	02:01:01G			
HLA B Set 1	44:DUUNB			
HLA B Set 2	57:DUUNC			
HLA C Set 1	05:01:01G			
HLA C Set 2	05:01:01G			
HLA DPA1 Set 1	07:01:01G			

Additional Information:
These cells were isolated from donated human tissue after obtaining permission for their use in research applications by informed consent or legal authorization. This product is for research use only. Details concerning the use of our cell and media products can be downloaded from our website at www.lonza.com/cell-protocols.

In addition to the specifications listed above, the following are guaranteed for all lots of this product using Lonza' s Clonetics (TM) and Poietics (TM) Media, Reagents, and Protocols: Total Population Doublings >=10.

This lot has been reviewed by Quality Assurance in compliance with requirements of Lonza' s Quality System. This document was generated from a validated Part 11-compliant electronic system and thus handwritten signatures are not required.

Lonza Cologne GmbH
Nattermannallee 1
D-50829 Cologne

For Technical Assistance, call 1-800-521-0390

Certificate of Analysis

eShop Dummy Customer SE
used in eShop only
SE-

Print Date: 27-Apr-2026

Product Name:	HSMM-Muscle Myoblasts SkGM-2, cryo amp
Material Number:	CC-2580
Batch No:	21TL138913
Manufacturing Date:	27-Jun-2021

Test	*RESULT*	*SPECIFICATION MIN*	*MAX*	*UNIT*

Cheryl Kitchen
Electronically signed by Cheryl Kitchen
Date: 09-AUG-2021 15:08:28 EST
Release (Inspection Lot: Usage Decision)

This lot has been reviewed by Quality Assurance in compliance with requirements of Lonza's Quality System. This document was generated from a validated Part 11-compliant electronic system and thus handwritten signatures are not required.

For Technical Assistance, call 1-800-521-0390

Lonza Cologne GmbH
Nattermannallee 1
D-50829 Cologne
